# Draft Genome Sequence for a Clinical Isolate of Vancomycin-Resistant *Enterococcus faecalis*

**DOI:** 10.1128/genomeA.00584-16

**Published:** 2016-06-23

**Authors:** Keesha E. Erickson, Nancy E. Madinger, Anushree Chatterjee

**Affiliations:** a Chemical and Biological Engineering, University of Colorado Boulder, Boulder, Colorado, USA; b Division of Infectious Diseases, University of Colorado Anschutz Medical Campus, Aurora, Colorado, USA; c BioFrontiers Institute, University of Colorado Boulder, Boulder, Colorado, USA

## Abstract

We report here the draft genome sequence of a multidrug-resistant *Enterococcus faecalis* strain, isolated from a patient at the University of Colorado Hospital. The genome assembly is 3,040,186 bp in length with 37.6% GC content. This isolate encodes eleven resistance genes, including those for glycopeptide, aminoglycoside, macrolide-lincosamide-streptogramin, and tetracycline resistance.

## GENOME ANNOUNCEMENT

Vancomycin-resistant enterococci (VRE) continue to pose a great public health threat, particularly to hospitalized individuals. VRE are estimated to lead to more than 20,000 infections and 1,300 fatalities annually in the United States alone (1). One common VRE is *Enterococcus faecalis*, which has been observed to be resistant to a multitude of antibiotics beyond glycopeptides (2). *E. faecalis* is recognized for encoding highly mobile elements (3–5), which enable rapid adaptation to antibiotic exposure. Expanding the library of *E. faecalis* genome sequences, particularly from multidrug-resistant clinical isolates, will allow for a better understanding of the dynamic nature of resistance in VRE infections and provide insights for future treatment.

The sequenced *E. faecalis* strain CU0714 was obtained from an organism bank maintained by the University of Colorado Hospital Clinical Microbiology Laboratory. The organism was recovered from the blood of a 44-year-old female patient with a central line-associated bloodstream infection on hospital day 222. Three weeks before the infection she had completed a 14-day course of vancomyin.

CU0714 colonies were suspended in cation-adjusted Mueller-Hinton broth (CAMHB) and incubated for 16 h under aerobic conditions at 37°C. DNA was isolated with the Wizard DNA purification kit (Promega). Lysis was performed with 120 µL of 30 mg/mL lysozyme and 60 µL of 10 mg/mL lysostaphin. Approximately 2 µg of DNA was submitted for sequencing. A paired-end 250-bp library was prepared with the Nextera XT DNA library kit and sequenced on an Illumina MiSeq platform, generating 542,058 reads. FASTQ files were filtered for quality using the sliding window mode in Trimmomatic (6). Paired reads were used for *de novo* assembly with SPAdes version 1.0.0 (7), Rescaf version 1.0.1, and annotation with PROKKA version 1.0.0 (8), all run via BaseSpace (https://basespace.illumina.com).

The draft genome sequence has a total size of 3,040,186 bp with 37.6% GC content, sequenced to 42× coverage. The assembly contains 69 contigs with a contig *N*_50_ of 220,545 bp. Approximately 42 tRNAs, 6 rRNAs, 1 orphan CRISPR2 array (9), and 2,905 coding sequences were identified. We used BLAST (10) to compare this assembly to others in NCBI’s genome database. Various contigs from *E. faecalis* CU0714 had >98% alignment/query coverage to strains, including B2949, B4270, Merz96, B337, and Ned10. PlasmidFinder (11) and BLAST revealed that CU0714 carries a conjugation plasmid similar to pCF10 (12); 78% of the pCF10 sequence was located with greater than 86% similarity.

We applied ARG-ANNOT (13) to locate antibiotic resistance genes. *E. faecalis* CU0714 encodes an estimated 11 resistance genes, including seven vancomycin (*vanS*, *vanB*, *vanH*, *vanW*, *vanY*, *vanR*, *vanX*), two macrolide-lincosamide-streptogramin (*lsaA*, *mphD*), one tetracycline (*tetM*), and one gentamicin (*aac6-aph2*). *tetM* is located on the conjugation plasmid, while other resistance genes appear to be encoded in the genome. Resistances were verified by plating cultures on CAMHB agar with antibiotics. CU0714 grew on tetracycline, gentamicin, vancomycin, and sulfadimidine, and is also resistant to ciprofloxacin. As there were no fluoroquinolone resistance genes identified, mutations in DNA gyrases and topoisomerases likely impart ciprofloxacin resistance (14). CU0714 is sensitive to ampicillin, meropenem, rifampin, erythromycin, and chloramphenicol.

### Nucleotide sequence accession number.

This whole-genome shotgun sequencing project has been deposited in GenBank under accession number LXID00000000.
